# Evaluation of Weight Loss Failure, Medical Outcomes, and Personal Experiences after Roux-en-Y Gastric Bypass: A Critical Analysis

**DOI:** 10.1155/2013/943423

**Published:** 2013-02-14

**Authors:** Rogier Hörchner, Dave Schweitzer

**Affiliations:** ^1^Ra-Medical Obesity Centre Beverwijk, Parallelweg 124-04, 1948 NN Beverwijk, The Netherlands; ^2^Department of Internal Medicine and Endocrinology, Reinier de Graaf Group of Hospitals, Reinier de Graafweg 3-11, 2625 AD Delft, The Netherlands

## Abstract

*Background*. Roux-en-Y gastric bypass (RYGB) is considered an effective and well-tolerated surgical procedure. In this retrospective study, we critically assessed efficacy and negative personal experiences (NPEs) after RYGB with a self-administered questionnaire (SAQ). *Methods*. This questionnaire study included 404 patients who had undergone RYGB. Analysis was performed using data from medical records, referral letters, and SAQs at an average of 33 months after procedure. We evaluated the occurrence of hypertension, CPEP use and type 2 diabetes mellitus (T2DM), the amount of excess weight loss, degree of satisfaction and negative personal experiences (NPEs) related to the procedure, and adherence to a dedicated life style program and (non)attendance to followup. consults after surgery. *Results*. 42.3% of all SAQs were evaluable for analysis. T2DM remained similar, while hypertension and continuous positive airway pressure (CPAP) use decreased significantly; excess weight loss of ≥40% was reported in 69% and of <40% in 19%, a significant improvement. Absolute weight gain was reported in 10.5%, fatigue in 44.4%, dysphagia in 11.6%, and other NPEs in 7.6%. Dissatisfaction over weight loss was reported in 9.4%. Mean number of follow-up visits was 9.6 per respondent, while nonattendance of any follow-up visit consults occurred in 1.8%. *Conclusions*. The use of post-RYGB SAQs provided evaluable data in 42.3%. Treatment failure after RYGB appears to be relevant, encouraging the use of SAQ studies in large cohorts.

## 1. Introduction

Morbid obesity is defined as a chronic condition for which consistent and durable changes of lifestyle are required. Improvement of life style remains critical in any treatment strategy, be it the medical or the surgical approach. In addition, multidisciplinary combined intervention programs for improving life style are mandatory according to international consensus [1].

The Roux-en-Y gastric bypass (RYGB) procedure has emerged as an effective treatment for morbid obesity, in particular for patients with metabolic syndrome and/or type 2 diabetes mellitus (T2DM), obstructive sleep apnea, and hypertension [2–6]. It has also been shown clearly that RYGB is both clinically beneficial and cost effective in the long run [2, 6]. The efficacy of bariatric surgery stems from long-lasting effects leading to a lower obesity-related morbidity and to a significant reduction of mortality [7]. However, weight regain after bariatric surgery diminishes the beneficial effects of surgical interventions as it may lead to recurrence of metabolic consequences such as T2DM, hypertension, dyslipidemia, and obstructive sleep apnea [8–11]. Therefore, one of the greatest challenges after bariatric surgery is to obtain and maintain a weight reduction by at least 40% excess weight loss (EWL) without drastic dietary interventions or postprandial complaints. It is generally believed that this approach is crucial to preserve metabolic health [12] but also to optimize quality of life. We investigated, using a questionnaire, whether accepted goals were appropriately achieved in a group of operated patients.

## 2. Patients and Methods

This questionnaire study aimed to analyze changes in hypertension, use of CPEP for obstructive sleep apnea, EWL, T2DM, satisfaction with weight loss achieved, negative personal experiences (NPEs), and number of/attendance to follow-up visits. The study was conducted at Ra-Medical Obesity Center Beverwijk, Beverwijk, The Netherlands. A multidisciplinary team consisting of surgeons, physicians, dieticians, nurse practitioners, psychologists, and anesthetists screened morbidly obese patients for bariatric surgery. Patients as well as their relatives were encouraged to attend a dedicated multidisciplinary aftercare program. Included were females and males between 18 and 65 years who met the criteria of the North American Association for the Study of Obesity (NAASO) and the International Federation for the Surgery of Obesity and Metabolic Disorders (IFSO), [13–15]. RYGBs were performed by 4 specialized bariatric surgeons in three different hospitals in The Netherlands and Belgium.

A total of 404 structured self-administered questionnaires (SAQs) were sent to 331 females and 73 males who had undergone surgery between March 01, 2004 and February 17, 2010. The questionnaire addressed 9 items: body weight, T2DM, hypertension, CPEP use, vitamin B12 levels, satisfaction with weight loss achieved, NPEs, and number of/attendance to follow-up visits (Table 1). One hundred and seventy-one SAQs (42.3%) were returned at a median postoperative period of 25 months (mean 33 months) and were usable for final analysis. According to protocol, each SAQ was cross checked for ID~confirmation and each response in the SAQ was compared with filed data (referral notes, medical records, and laboratory results). T2DM was defined as a fasting blood glucose (FBG) >7.0 mmol/L and/or a glycated hemoglobin (HbA_1c_) >53 mmol for at least 4 years including the use of oral antidiabetics on a daily basis. These data were verified in the medical charts of Ra-medical Obesity Center Beverwijk, written at the moment of referral. Hypertension was defined as a blood pressure of >140/90 mmHg either with or without use of oral antihypertensives since at least 4 years, verified in the medical charts at the moment of referral to Ra-medical Obesity Center, written at the moment of referral. SAQ items were subdivided, that is, into satisfaction about weight loss (SWL), surgical complications (SCs) and negative personal experiences. Micronutrient deficiencies reported in the SAQ were cross-checked with existing laboratory data. All 171 respondents had participated in the Ra-Medical aftercare Program. A nurse practitioner (NP), a psychologist, and a dietician took part in this multidisciplinary program and individual visits were planned at 3-month intervals.

### 2.1. Statistical Analysis

SPPS 17.0 (SPSS Inc., Chicago, IL, USA) software was used for statistical analysis. Variables are expressed as mean ±1 standard deviation (SD) and analyzed with paired Student-tests (two-sided). A two-sided *P* value of 0.01 was used as threshold for statistical significance. 

## 3. Results 

Females (145/331) responded, were 44 ± 11 (range: 21–65) years of age, body weight before surgery 140.7 ± 23.8 kg (overal), BMI 49.9 kg/m² versus (after surgery) 99.7 ± 20.9 kg, (*P* < 0.01), BMI 35.3 kg/m² (*P* < 0.01) with an EWL of 69%. Males responded, (26/73) were 46 ± 9 (range: 20–59) years of age, body weight before surgery 167.9 ± 28.9 kg, BMI 50.9 kg/m² versus (after surgery) 109.8 ± 21.4 kg (*P* < 0.01), BMI 33.2 kg/m² (*P* < 0.01) with an EWL of 70%. Disappointment about weight loss was reported in 43 patients (25.2%) and EWL ≤ 40% in 29 patients (19.0%). 18 out of 171 patients (10.5%) reported absolute weight gain. EWL > 40% was reported in 142/171 patients (83.0%). All patients who experienced absolute weight gain underwent a hybrid gastric pouch restriction with a gastric band, carried out as a result of the lack of weight loss. T2DM was present in 30 patients (17.5%) before surgery and in 16 patients after surgery (9.3%, *P* = 0.357); 14 patients (8.2%) received oral antidiabetic drugs and 2 patients were on insulin (Table 2). 

Hypertension was present before surgery in 51 patients (29.8%) and had decreased after surgery to 23 patients (13.5%) (*P* < 0.01), each of them receiving antihypertensive drugs. Ten out of 171 patients were CPEP users before surgery, versus 1 after (*P* < 0.01).

Completed SAQs were received from 145 of 331 females (43.8%) and from 26 of 73 males (35.6%). For each SAQ, the ID agreed with that noted in the medical record of the respondent.

All respondents reported full adherence to the aftercare program, and attendance numbers were equal between genders: 9.6 ± 5.0 (range in females 1–31 and in males 1–21). The distribution of SAQ number per year of surgery is listed in Table 3.

Surgical complications were reported in 13 SAQs (6 females and 7 males); each patient's report matched with the medical record (Table 4). None of the respondents were smokers or heavy drinkers according to SAQ and verified in the medical records of Ra-medical Obesity Center Beverwijk. 

Satisfaction about weight loss (SWL) was reported as “above expectation” by 51 patients (29.8%)—37 females and 14 males, “good” by 71 patients (41.5%)—62 females and 9 males, “adequate” by 33 patients (19.3%)—32 females and 1 male, and “poor” by 16 patients (9.4%)—14 females and 2 males. In addition to SWL, open-ended questions about negative thoughts or feelings (NPEs) as well as memorized and verified micronutrient deficiencies are listed in Table 5. NPEs were reported by 160 patients (136 females, 24 males); 8 females and 1 male reported more than 3 NPEs. Dysphagia occurred in 20 respondents (11.6%)—18 females and 2 males.

There was no correlation between SWL and surgical complications (SCs), see Table 6. SCs were reported by 6 females and 7 males; 3 of these patients reported SWL as “less than expected.” On the contrary, 10 of 13 patients with SCs reported SWL as “sufficient,” “good” or “above expectation” (Table 6). 

## 4. Discussion 

The present study focused on the efficacy of RYGB in optimizing health, and also considered personally relevant but infrequently reported side effects. To this end, we studied a heterogeneous group of patients who were requested to respond to a self-administered questionnaire at a mean of 33 months (median 25) after surgery. The response was 171 out of 404 SAQs (42%)—145 of 331 females (43%) and 26 of 73 males (36.0%). This response might seem to be rather low at first sight, but it is still considered to be solid in resolving emerging clinical research questions [16]. Importantly, this questionnaire study showed significant improvements of hypertension and CPEP use but no significant improvement of T2DM prevalence according to the definition as used in the protocol of this research (*P* = 0.357). 

Some limitations of this type of surgery clearly need further examination, in particular the phenomenon of disappointing weight reduction or even weight increase over time and patients complaints about fatigue and dysphagia. Weight reduction is the cornerstone of efficacious treatment for patients and surgeons. Weight reduction is thought to result from the combined effects of food restriction, malabsorption [17–20], and changes in neurointestinal regulation that cause appetite suppression [21, 22]. However, Swedish investigators specifically looking for neurointestinal regulation one year after surgery discovered a reset of Ghrelin production back to preoperative levels [23]. Moreover, research in rodents has shown expansion of villous mass after gastric bypass and duodenal switch, thereby enhancing the intestinal resorption capacity [24, 25]. The adaptive capacity of the gut has also been shown in the clinic. Overweight and mildly obese patients who received a biliopancreatic diversion (partially) recovered from T2DM and aspects of the metabolic syndrome with a relatively moderate weight reduction up to a level that malnourishment did not occur [26]. Still, the issue of variability of weight reduction after bariatric procedures remains incompletely understood since validated prognostic weight markers at baseline are lacking. 

The complaint of fatigue deserves more scientific attention because of its high prevalence in the clinic. In the present study, 40 out of 136 females (29.0%) and 7 out of 24 males (29.0%) complained of fatigue after surgery. Obviously, clinicians must always stay alert for some well-known causes of fatigue, such as nutritional deficiencies and weight gain. In this study, we found insufficient or deficient vitamin B12 levels in 44.4% of patients, while vitamin B12 deficiency has previously been reported to be 33.0% at 2 years after RYGB [17]. Anecdotally, fatigue can completely disappear with vitamin B12 supplementation; however, the level of evidence is too weak to use vitamin B12 as a panacea for the complaint of fatigue after surgery.

This study was not designed to study weight loss related to quality of life (QoL), hypertension, CPEP-use, T2DM as well as satisfaction, negative personal experiences (NPEs) and followup (non)attendance after surgery. Recently, two validated scoring tools—the Nottingham Health Profile and the Bariatric Analysis and Reporting Outcome System (BAROS)—have been shown to be useful in assessing QoL [27]. Specific gastrointestinal scoring tools for food tolerance and gastrointestinal QoL have been shown to have sufficient power in discriminating QoL after adjustable gastric banding, RYGB, and sleeve gastrectomy [28, 29]. Notably, the SAQ used in the current study included several close-ended as well as open-ended questions to score the respondent's personal views and opinions about the results of surgery. Moreover, close-ended questions like “do you have hair loss” or “do you feel tired” were intentionally avoided to minimize investigator bias. To our knowledge, there are no data in the medical literature onpersonal views and complaints with regard to a past bariatric treatment.

Notably, we found a complete match between NPEs mentioned in the SAQs and notes in the medical record of each patient, which favors the strength of the data. However, only 43.0% of females and 36.0% of males returned an evaluable SAQ. This may have weakened the study. Therefore, prospective questionnaire studies need to be conducted with short intervals so as to be able to encourage those patients who otherwise may get lost to followup.

In conclusion, EWL ≤ 40.0% appears to be a common phenomenon after RYGB [19] and may be a plausible factor in persistence of the metabolic syndrome, fatigue, and NPEs. More research is needed to study weight (re)gain and to discover treatable factors to prevent treatment failure after bariatric surgery. 

## Figures and Tables

**Table 1:** Items used in the self administered questionnaire (SAQ).

Question	Item structure
(1) Satisfaction with weight loss	Close-ended
(i) Above expectation	
(ii) Good	
(iii) Sufficient	
(iv) Less than expected	

(2) Weight loss	Closed-ended compared with medical records

(3) Did you make an appointment at the website for the aftercare program?	Closed-ended compared with medical records

(4) Do you want to make a new appointment for the aftercare program with a:	Closed-ended compared with medical records
(i) Nurse practitioner	
(ii) Dietician	
(iii) Mental coach	

(5) Do you want to make a new appointment for a group session?	Closed-ended compared with medical records

(6) I want to make an appointment for the aftercare Program. My motivation is:… …	Open-ended

(7) Age	Closed-ended

(8) Gender	Closed-ended

(9) Negative personal experiences	Open-ended

Diabetes	Information from medical records
Hypertension	Information from medical records

**Table 2:** Prevalence of T2DM, hypertension and obstructive sleep apnea before and after surgery in females and males.

	T2DM			Hypertension			Use of CPEP		
	Pre surg.	Post surg.	*P* value	Pre surg.	Post surg.	*P* value	Pre surg.	Post surg.	*P* value
Females	22 (145)	13 (145)	0.620	39 (137)	17 (137)	<0.01	6 (145)	0 (145)	<0.01
Males	8 (25)	3 (25)	0.454	12 (24)	6 (24)	<0.01	4 (25)	1 (25)	<0.01

*P* values represent comparisons of T2DM, hypertension and CPEP use before and after surgery in females and males. Available data from patients who returned SAQs are written between parentheses.

**Table 3:** SAQ's returned per year of surgery, consultation numbers, and excess weight loss expressed as means.

Year of surgery	Females			Males		
	SAQs	Consultations	EWL %	SAQs	Consultations	EWL %
2004	4	15	27	0	—	—
2005	7	7.7	63	1	7.0	68
2006	14	9.9	59	6	11.7	69
2007	19	7.9	57	2	17.5	62
2008	31	10.6	67	6	9.8	67
2009	62	10.2	59	10	7.7	76
2010	8	8.1	49	1	1.0	67

**Table 4:** Surgical complications.

Surgical complications (SCs)	Female (*n* = 145)	Male (*n* = 26)	(%) of 171 included patients
Postoperative anastomotic leakage	1	3	2.3
Adhesions	2	2	2.3
Ulcera at the gastrojejunostomy	2	2	2.3
(Troicart) Hernia abdominalis (abdominal wall)	1	—	0.6

**Table 5:** Negative personal experiences (NPEs) after RYGB.

Item	No. of SAQs with NPE (*n* = 160)		
	Female (*n* = 136)	Male (*n* = 24)	Percent (*n*/171)
I think I have regular dumping attacks	7	6	7.6
I feel tired (fatigue)	67	9	44.4
I have regular cramps in my abdomen	9	4	7.6
I have regular difficulties with swallowing	18	2	11.7
I feel regular heartburns	2	3	2.9
I think my blood glucose is frequently low	1	2	1.8
I belch regularly	2	2	2.3
I feel nauseous every day	10	2	7.0
My stools are too watery	5	2	4.1
I am regularly constipated	6	1	4.1
I have intolerance for milk and milk products	2	1	1.8
I suffer from sleeplessness	1	—	0.6
I feel depressed after the operation	2	—	1.2
I often have food cravings	2	3	2.9
I have hair loss	3	—	1.8
I have joint pain	4	1	2.9
I have regular itching	1	1	1.2
I sweat a lot	1	—	0.6
I regularly crave for a large meal	7	4	6.4
In my view there is an urgent need for an abdominoplasty	8	3	6.4

	Micronutrient deficiencies reported in SAQ and confirmed in the medical records		

Potassium	—	1	0.6
Calcium and/or vitamin D	6	1	4.1
Iron	9	—	5.3
Vitamin K	1	—	0.6
Folic acid	1	—	0.6
Vitamin B12^a^	67	9	44.4

^a^Vitamin B12 deficiency according to SAQ was verified as either insufficiency (levels of 150–220 nmol/L) or deficiency (levels <150 mmol/L).

**Table 6:** Satisfaction with weight loss compared to complication.

					Satisfaction with weight loss					
Surgical complications	Number of occurrences		Above expectation		Good		Sufficient		Less than expected	
	Female	Male	Female	Male	Female	Male	Female	Male	Female	Male
Postoperative anastomotic leaks	1	3	1	—	—	2	—	—	—	1
Adhesions	2	2	—	1	—	1	1	—	1	—
Ulcers at the gastrojejunostomy	2	2	—	—	2	1	—	1	—	—
(Troicart) Hernia abdominalis (abdominal wall)	1	—	—	—	—	—	—	—	1	—

## References

[1] Apovian CM, Cummings S, Anderson W (2009). Best practice updates for multidisciplinary care in weight loss surgery. *Obesity*.

[2] Pories WJ, Swanson MS, MacDonald KG (1995). Who would have thought it? An operation proves to be the most effective therapy for adult-onset diabetes mellitus. *Annals of Surgery*.

[3] Pories WJ (2011). The IDF statement: a big and long-awaited step for our diabetic patients. *Obesity Surgery*.

[4] Huang CK, Shabbir A, Lo CH (2011). Laparoscopic Roux-en-Y gastric bypass for the treatment of type II diabetes mellitus in Chinese patients with body mass index of 25–35. *Obesity Surgery*.

[5] Buchwald H, Estok R, Fahrbach K (2009). Weight and type 2 diabetes after bariatric surgery: systematic review and meta-analysis. *American Journal of Medicine*.

[6] Sjöström CD, Peltonen M, Wedel H (2000). Differentiated long-term effects of intentional weight loss on diabetes and hypertension. *Hypertension*.

[7] Sjöström L, Narbro K, Sjöström CD (2007). Effects of bariatric surgery on mortality in Swedish obese patients. *The New England Journal of Medicine*.

[8] Shah M, Simha V, Garg A (2006). Review: long-term impact of bariatric surgery on body weight, comorbidities, and nutritional status. *Journal of Clinical Endocrinology and Metabolism*.

[9] Sugerman HJ, Wolfe LG, Sica DA (2003). Diabetes and hypertension in severe obesity and effects of gastric bypass-induced weight loss. *Annals of Surgery*.

[10] Sjöström CD, Peltonen M, Wedel H, Sjöström L (2000). Differentiated long-term effects of intentional weight loss on diabetes and hypertension. *Hypertension*.

[11] Pinkney J, Kerrigan D (2004). Current status of bariatric surgery in the treatment of type 2 diabetes. *Obesity Reviews*.

[12] Mathus-Vliegen EMH (2003). Nutrition and health—ideal body weight unrealistic; health benefit by moderate sustained weight loss. *Nederlands Tijdschrift voor Geneeskunde*.

[13] NAASO (2008). *The Practical Guide to the Identification, Evaluation, and Treatment of Overweight and Obesity in Adults*.

[14] National Institutes of Health, National Heart, Lung, and Blood Institute (1998). Clinical guidelines on the identification, evaluation, and treatment of overweight and obesity in adults-the evidence report. National Institutes of Health, National Heart, Lung, and Blood Institute. *Obesity Research*.

[15] Fried M, Hainer V, Basdevant A (2007). Inter-disciplinary European guidelines on surgery of severe obesity. *International Journal of Obesity (London)*.

[16] Holbrook AL, Krosnick JA, Pfent A (2007). The causes and consequences of response rates in surveys by the news media and government contractor survey research firms. *Advances in Telephone Survey Methodology*.

[17] Vargas-Ruiz AG, Hernández-Rivera G, Herrera MF (2008). Prevalence of iron, folate, and vitamin B12 deficiency anemia after laparoscopic Roux-en-Y gastric bypass. *Obesity Surgery*.

[18] Shah M, Simha V, Garg A (2006). Review: long-term impact of bariatric surgery on body weight, comorbidities, and nutritional status. *Journal of Clinical Endocrinology and Metabolism*.

[19] Campos GM, Rabl C, Mulligan K (2008). Factors associated with weight loss after gastric bypass. *Archives of Surgery*.

[20] DiGiorgi M, Daud A, Inabnet WB (2008). Markers of bone and calcium metabolism following gastric bypass and laparoscopic adjustable gastric banding. *Obesity Surgery*.

[21] Bose M, Oliván B, Teixeira J, Pi-Sunyer FX, Laferrère B (2009). Do incretins play a role in the remission of type 2 diabetes after gastric bypass surgery: what are the evidence?. *Obesity Surgery*.

[22] Ybarra J, Bobbioni-Harsch E, Chassot G (2009). Persistent correlation of ghrelin plasma levels with body mass index both in stable weight conditions and during gastric-bypass-induced weight loss. *Obesity Surgery*.

[23] Pérez-Romero N, Serra A, Granada ML (2010). Effects of two variants of Roux-en-Y gastric bypass on metabolism behaviour: focus on plasma ghrelin concentrations over a 2-year follow-up. *Obesity Surgery*.

[24] Borg CM, Roux CWL, Ghatei MA, Bloom SR, Patel AG (2007). Biliopancreatic diversion in rats is associated with intestinal hypertrophy and with increased GLP-1, GLP-2 and PYY levels. *Obesity Surgery*.

[25] Le Roux CW, Borg C, Wallis K (2010). Gut hypertrophy after gastric bypass is associated with increased glucagon-like peptide 2 and intestinal crypt cell proliferation. *Annals of Surgery*.

[26] Scopinaro N, Adami GF, Papadia FS (2011). Effects of biliopanceratic diversion on type 2 diabetes in patients with BMI 25 to 35. *Annals of Surgery*.

[27] Martínez Y, Ruiz-López MD, Giménez R (2010). Does bariatric surgery improve the patient's quality of life?. *Nutrición Hospitalaria*.

[28] Overs SE, Freeman RA, Zarshenas N (2012). Food tolerance and gastrointestinal quality of life following three bariatric procedures: adjustable gastric banding, Roux-en-Y gastric bypass, and sleeve gastrectomy. *Obesity Surgery*.

[29] Lier HO, Biringer E, Hove O (2011). Quality of life among patients undergoing bariatric surgery: associations with mental health- A 1 year follow-up study of bariatric surgery patients. *Health and Quality of Life Outcomes*.

